# miR-302b inhibits cancer-related inflammation by targeting ERBB4, IRF2 and CXCR4 in esophageal cancer

**DOI:** 10.18632/oncotarget.17041

**Published:** 2017-04-11

**Authors:** Mingxin Zhang, Lingmin Zhang, Manli Cui, Wenguang Ye, Pengjiang Zhang, Suna Zhou, Jingjie Wang

**Affiliations:** ^1^ Department of Gastroenterology, Tangdu Hospital, Fourth Military Medical University, Xi'an 710038, Shaanxi Province, China; ^2^ Department of Anesthesiology, First Affiliated Hospital, Xi'an Jiaotong University, Xi'an 710061, Shaanxi Province, China; ^3^ Second Department of Cadre's Ward, Lanzhou General Hospital of Chinese PLA, Lanzhou 730050, China; ^4^ Department of Radiotherapy, Tangdu Hospital, Fourth Military Medical University, Xi'an 710038, Shaanxi Province, China

**Keywords:** miR-302b, cancer related inflammation, esophageal cancer

## Abstract

Cancer related inflammation (CRI) plays an important role in the development of esophageal cancer (EC), and the target gene analysis shows that miR-302b potential target genes closely correlated to CRI important signaling pathways. The present study was to evaluate the inhibition of miR-302b on CRI in EC and its mechanism. We found that the expression levels of miR-302b in EC cells were lower than that in Het-1A cells, while TE11 with the lowest expression and OE33 with the highest. Inflammatory stimuli at 48 h significantly reduced expression of miR-302b in EC cells, but had no effect in Het-1A. After up-regulation of miR-302b in TE11 and down-regulation of miR-302b in OE33, it was found that miR-302b reduced CRI key transcription factors and representative cytokines. Then, over-expressed of miR-302b significantly altered potential target genes protein expressions and there was a negative correlation between miR-302b and potential target genes protein expressions (ERBB4, IRF2 and CXCR4) in EC tissues. Then reporter gene analysis revealed that miR-302b post-transcriptionally regulated expression of target genes by specific area of 3′-UTR. Transfected by target genes shRNA plasmids together could get the same effects of miR-302b on protein expression of CRI key transcription factors. Furthermore, miR-302b was able to repress tumor growth and transcription factors protein expression *in vivo*. These finding suggests that miR-302b inhibits key transcription factors and cytokines by targeting ERBB4, IRF2 and CXCR4, implicating its role in the inhibition of CRI in EC.

## INTRODUCTION

Esophageal cancer (EC) is the eighth most common cancer worldwide, and the sixth most common cause of death from cancer [1]. There are two main histological types of EC, including esophageal adenocarcinoma (EAC) and esophageal squamous cell carcinoma (ESCC). China contributed almost half of the global new esophageal cancer cases in 2012, and most of these cases were pathologically confirmed as ESCC [2]. Despite a myriad of improvements in both diagnostic and therapeutic techniques over the past three decades, EC still has a poor prognosis, meaning that EC is a major public health problem in China [3]. It calls for more and more research on the molecular mechanisms responsible for EC carcinogenesis and development.

Recently, cancer-related inflammation (CRI) has been proposed as a major physiological hallmark of malignancy. Chronic infection and inflammation contribute to about 25% of all cancer cases worldwide, and patients with chronic inflammation are more susceptible to develop cancer [4]. Extensively investigations have revealed the key mediators of CRI, including transcription factors, chemokines, cytokines, and so on. These mediators show important value in diagnosis, treatment, and prognosis in different types of cancer. Our previously review focus on the role CRI in EC and found that CRI critical factors in EC are transcription factor NF-κB, STAT3 and HIF-1α and their downstream cytokines such as IL-6, IL-23, and TNF-β. But the regulation mechanisms of CRI in EC are unclear [5].

MicroRNAs (miRNAs) are small noncoding RNAs and post-transcriptionally regulate lots of genes involved in metabolism, age, and cancer [6]. Our previous study found that miR-302b was a potential molecular marker and inhibited proliferation by inducing apoptosis and repressed invasion potentially by targeting ErbB4 in ESCC [7]. miR-302b also down-expressed in other tumors such as breast cancer, gastric cancer, and hepatocellular carcinoma by targeting different target genes, suggesting that miR-302b functioned as a tumor suppressor in cancer [8–10]. But the exact mechanisms of miR-302b in cancer called for further research. Using the bioinformatics database, IRF2 and CXCR4 are predicted to be targets of miR-302b. Through literatures review, we found that ERBB4, IRF2 or CXCR4 has cross-talk with HIF-1α, STAT3, and NF-κB respectively [11–13]. As was mentioned above, we hypothesis that miR-302b may inhbit CRI in EC by targeting multiple genes closely related to CRI.

In this study, we first found down-expression of miR-302b in EC cell lines and tissues, and inflammation stimuli could change the expression levels. Then, increased or decreased expression of miR-302b could inhibit or promote CRI related critical pathway such as HIF-1α, STAT3, and NF-κB and cytokines such as IL-6, IL-23, and TNF-β. Furthermore, ERBB4, IRF2 and CXCR4 were confirmed as direct target genes of miR-302b and inhibition of these genes had the same effect of miR-302b on CRI. Last, *in vivo* experiments demonstrated that miR-302b functioned as a tumor suppressor by suppressing tumor growth, and more important was that CRI were also inhibited by miR-302b *in vivo*. These findings illustrated the tumor suppressor role of miR-302b in the control of CRI in EC, which might be a beneficial strategy for cancer therapy in future.

## RESULTS

### Down-expression of miR-302b in EC cells and tissues

**Figure 1:**
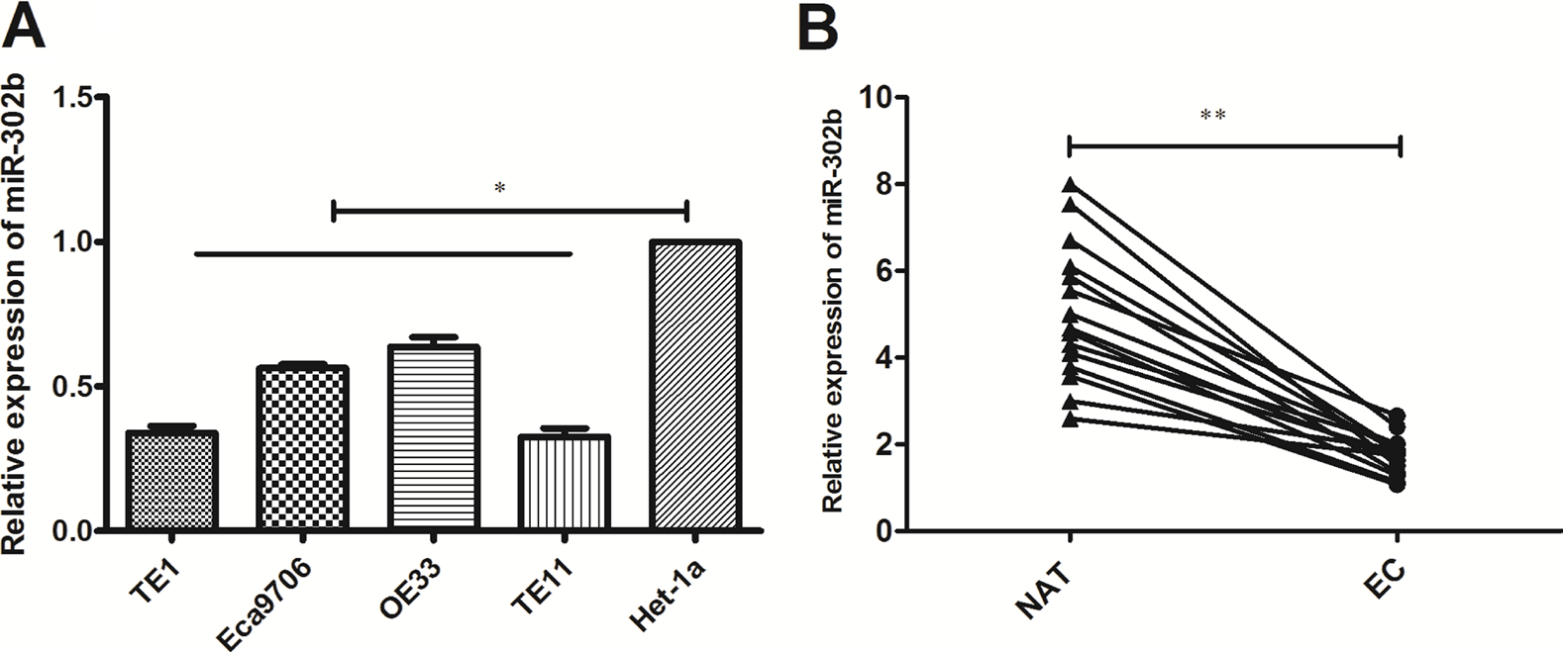
qRT-PCR of miR-302b expression in EC cells and tissue samples (**A**) qRT-PCR detected of miR-302b expression in EC cell lines (TE1, OE33, TE11 and Ec9706) and esaphagel normal cell line (Het-1a). (**B**) qRT-PCR detection of miR-302b expression in 15 paired EC and adjacent normal tissues. U6 was used as an internal control. Each assay was performed at least in triplicate. Corresponding *P* values determined by *t*-tests are indicated. EC: esophageal cancer tissues; NAT: adjacent normal tissues. *P < 0.05, **P < 0.01 vs control.

To explore the role of miR-302b in EC, we analyzed the expression of miR-302b in 4 EC cell lines (including 3 ESCC cell lines and 1 EAC cell line) by RT-PCR. Compared with esaphagel normal cell line (Het-1A), significant downregulation of miR-302b was observed in all EC cell lines, with TE11 cells expressing the lowest and OE33 cells expressing the highest (Figure 1A). We chose TE11 and OE33 for further experiments. Next, we found that miR-302b was downregulated in 15 paired esophageal cancer and adjacent noncancerous esophageal tissues (Figure 1B). Since we have reported the clinical and prognostic significance of miR-302b previously, we did not do further analysis.

### Decrease of miR-302b expression reacted to inflammation stimuli

**Figure 2:**
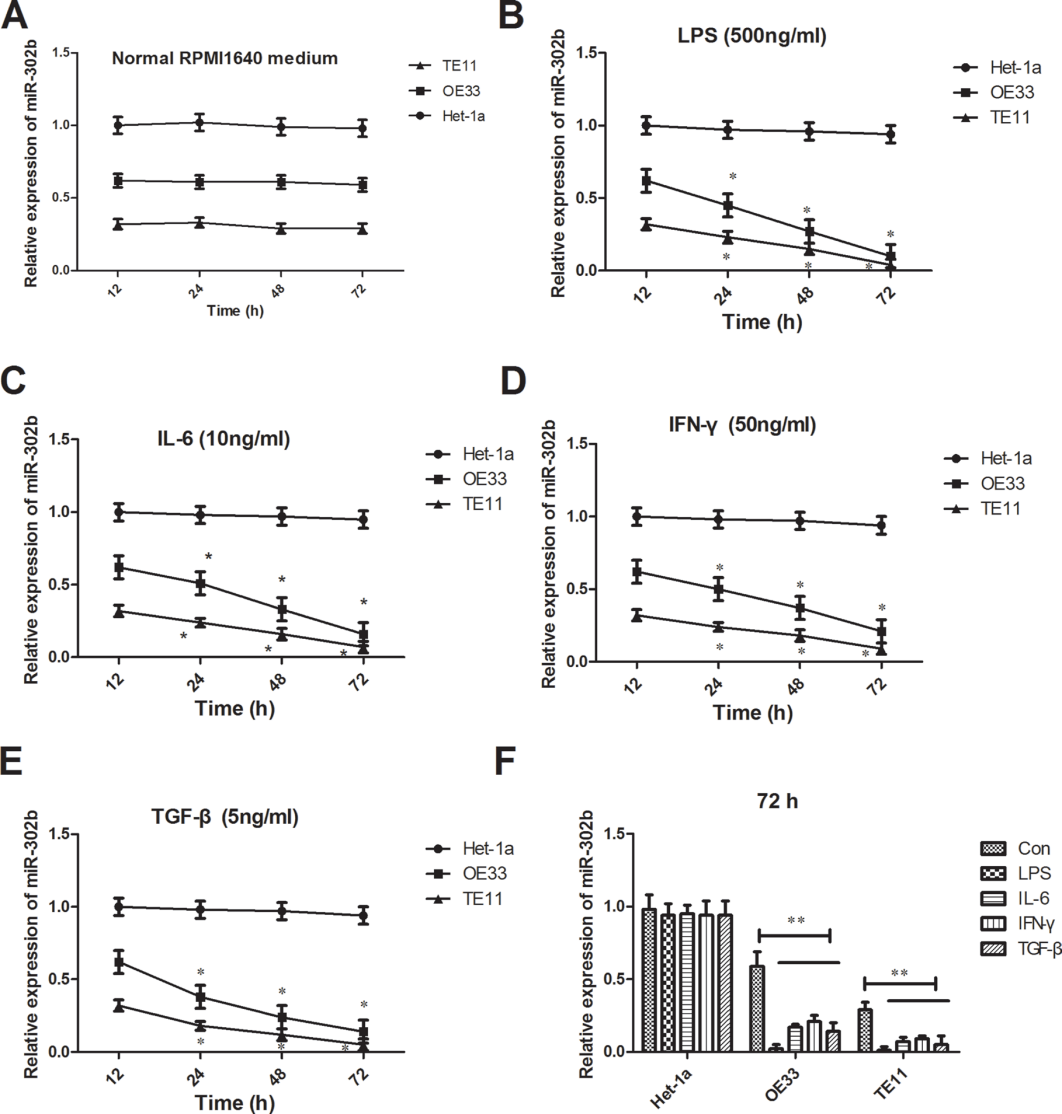
Changes of miR-302b expression reaction to inflammation stimuli (**A**) qRT-PCR detected miR-302b expression in TE11 and OE33 cells treated with normal RMPI1640 medium in 12 h, 24 h, 48 h, and 72 h. (**B**–**E**) qRT-PCR detected miR-302b expression in TE11 and OE33 cells treated with LPS (B), IL-6 (C), IFN-γ (D) and TGF-β (E) in 12 h, 24 h, 48 h, and 72 h. (**F**) qRT-PCR detected miR-302b expression in TE11 and OE33 cells treated with inflammation stimuli and normal medium in 72 h. U6 was used as an internal control. Each assay was performed at least in triplicate.*P < 0.05, **P < 0.01 vs control or Het-1a.

To investigate the role of miR-302b facing inflammation, we detected the changes of miR-302b in TE11 and OE33 reaction to different inflammation stimuli. The expression of miR-302b did no changes treated with normal RMPI1640 medium for 72 h, either in Het-1a, OE33 or TE11 (Figure 2A). But all the inflammation factors including LPS (500 ng/ml), IL-6 (10 ng/ml), IFN-γ (50 ng/ml) and TGF-β (5 ng/ml) down-regulated the expression of miR-302b in a time-dependent manner for both OE33 and TE11, while had no effect on Hel-1a (Figure 2B–2E). But there were no difference between two cell lines in all time points and also no differences among the four inflammation stimuli in 72 h (Figure 2F).

### miR-302b inhibits CRI *in vitro*

**Figure 3:**
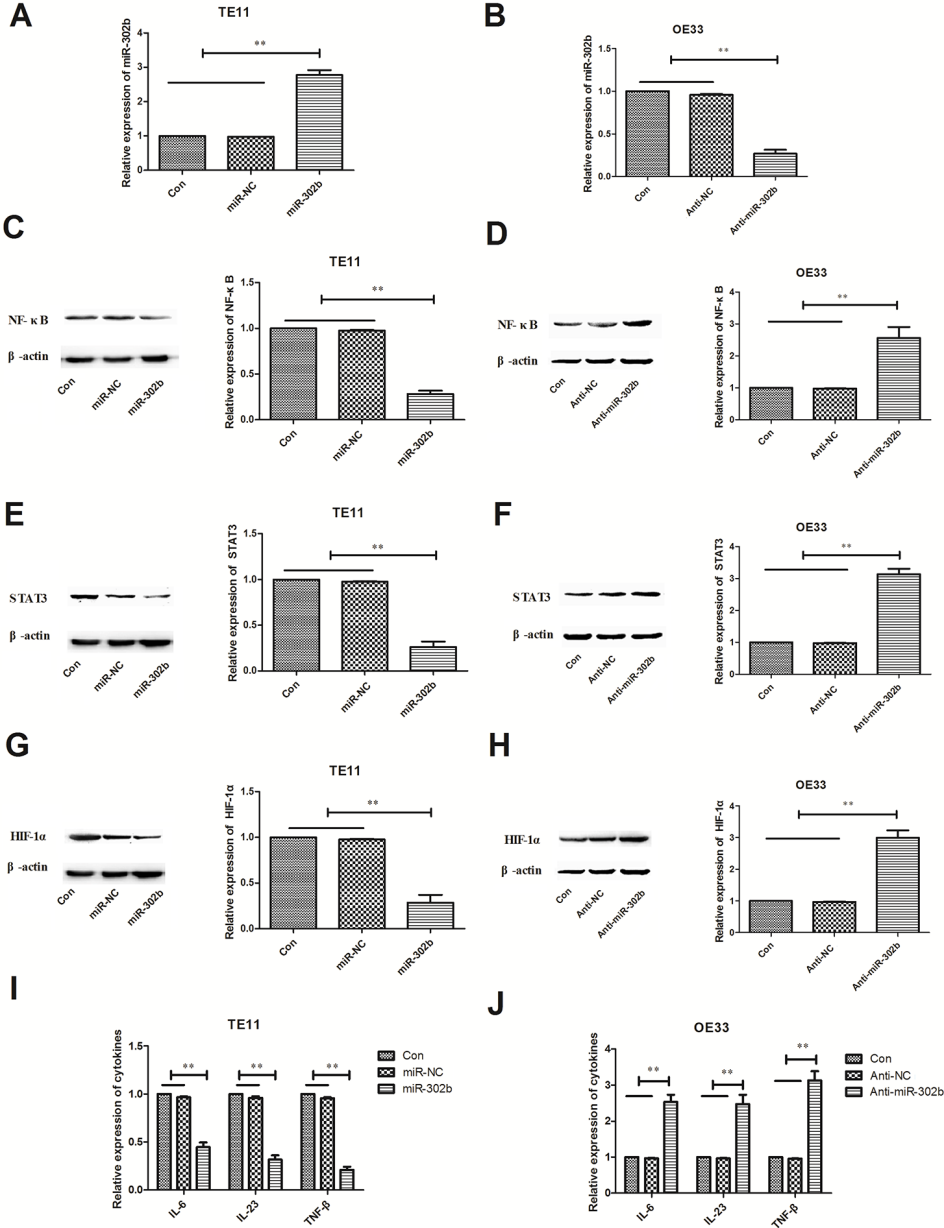
Effect of miR-302b expression on CRI critical pathway and cytokines (**A**) qRT-PCR detected miR-302b expression in TE11 cells transfected with mock (miR-NC) or miR-302 expression vector (miR-302b). (**B**) qRT-PCR detected miR-302b expression in OE33 cells transfected with mock (Anti-NC) or anti-miR-302b inhibitor sequence (anti-miR-302b). (**C**–**D**) Effect of up-regulation or down-regulation miR-302b on expression of NF-κB protein. (**E**–**F**) Effect of up-regulation or down-regulation miR-302b on expression of STAT3 protein. (**G**–**H**) Effect of up-regulation or down-regulation miR-302b on expression of HIF-1α protein. (**I**–**J**) Effect of up-regulation or down-regulation miR-302b on cytokines (IL-6, IL-23, and TNF-β) expression. U6 or β-actin was used as an internal control. Each experiment was performed at least in triplicate. *P < 0.05, **P < 0.01 vs control.

NF-κB, STAT3, and HIF-1α are important transcription factors playing critical role in CRI pathway, and regulate lots of inflammation factors such as cytokines expression. In this way, we further detected changes of key transcription factors (NF-κB, STAT3, and HIF-1α) and representative cytokines (IL-6, IL-23, and TNF-β) when modulating miR-302b expression in OE33 or TE11 cells. We up-regulated the miR-302b expression in TE11 cells by expression vectors and down-regulated it in OE33 cells by anti-miR sequence (Figure 3A–3B). Further experiments showed that over-expression of miR-302b decreased NF-κB, STAT3, and HIF-1α expression and certain cytokines in TE11 cells (Figure 3C, 3E, 3G, 3I), while inhibitation of miR-302b got the opposite results (Figure 3D, 3F, 3H, 3J).

### Targets of miR-302b are related to CRI

**Figure 4:**
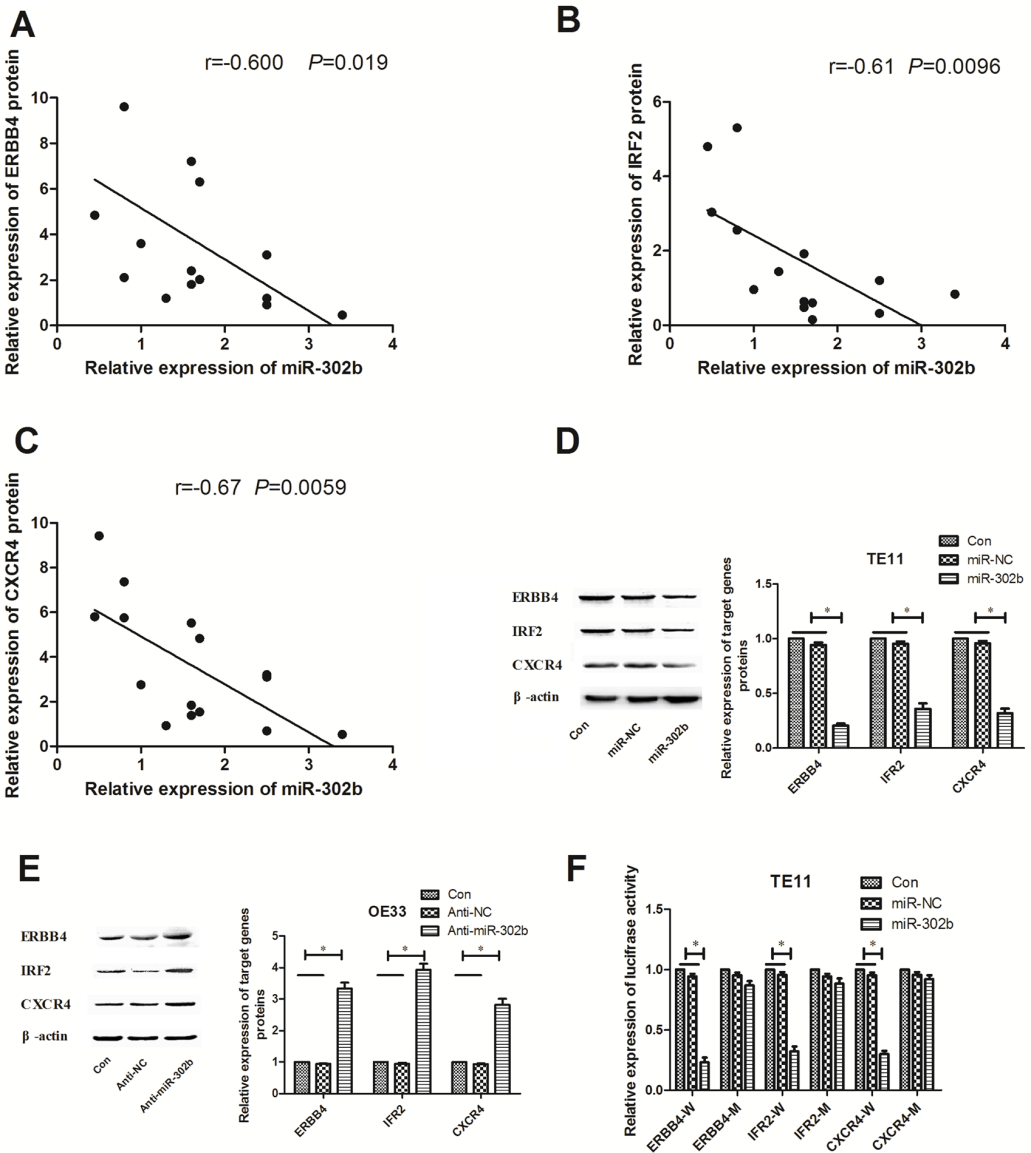
miR-302b targets ERBB4, IRF2 and CXCR4 by binding to the 3′-UTR region (**A**) Statistical analysis reveals an inverse correlation between relative miR-302b and IRF2 protin expression level in EC tissues. (**B**) Statistical analysis reveals an inverse correlation between relative miR-302b and ERBB4 protin expression level in EC tissues. (**C**) Statistical analysis reveals an inverse correlation between relative miR-302b and CXCR4 protin expression level in EC tissues. (**D**) Western blots detected of ERBB4, IRF2 and CXCR4 protein expression in TE11 cells transfected with mock (miR-NC) or miR-302 expression vector (miR-302b). (**E**) Western blots detected of ERBB4, IRF2 and CXCR4 protein expression in OE33 cells t transfected with mock (Anti-NC) or anti-miR-302b inhibitor sequence (anti-miR-302b). (**F**) pmirGLO luciferase vector contains wide type or mutant 3′-UTR of potential target genes and mock (miR-NC) or miR-302 expression vector (miR-302b) were co-transfected into TE11 cells, and cell lysates prepared at 48 h for measuring luciferase activity, which was normalized to Renilla luciferase activity. U6 or β-actin was used as an internal control. Each experiment was performed at least in triplicate. *P < 0.05, **P < 0.01 vs control.

Since miR-302b inhibited the CRI pathway, we then revealed the molecular mechanisms. To explore the role of miR-302b in regulation of CRI in EC development, we searched for potential target genes of miR-302b using miRNA databases (TargetScan, miRanda, and MiRDB), and putative miR-302b binding site was identified located within the 3′UTR of ERBB4, IRF2 and CXCR4. We first detected the correlation of miR-302b and these targets protein expression in EC tissues. As shown in Figure 4A–4C, all the targets expression negatively related to that of miR-302b in EC tissues. After up-regulation or down-regulation in TE11 cells or OE33 cells, expression of ERBB4, IRF2 and CXCR4 decreased or increased respectively (Figure 4D and Figure 4E). To further assess whether ERBB4, IRF2 or CXCR4 was a direct target of miR-320b, the luciferase reporter vector with the target genes 3′-UTR including the putative target site for miR-302b downstream of the luciferase genes (ERBB4-W/IRF2-W/CXCR4-W) and a mutant version (ERBB4-M/IRF2-M/CXCR4-M) were also constructed. Data indicated that miR-302b inhibited luciferase activity compared with the miR-NC group for ERBB4-W, IRF2-W, and CXCR4-W in TE11 cells, but had no effect in vector group containing mutant version (Figure 4F), suggesting that miR-302b interacts directly with the 3′-UTR of ERBB4, IRF2 and CXCR4 mRNA.

**Figure 5:**
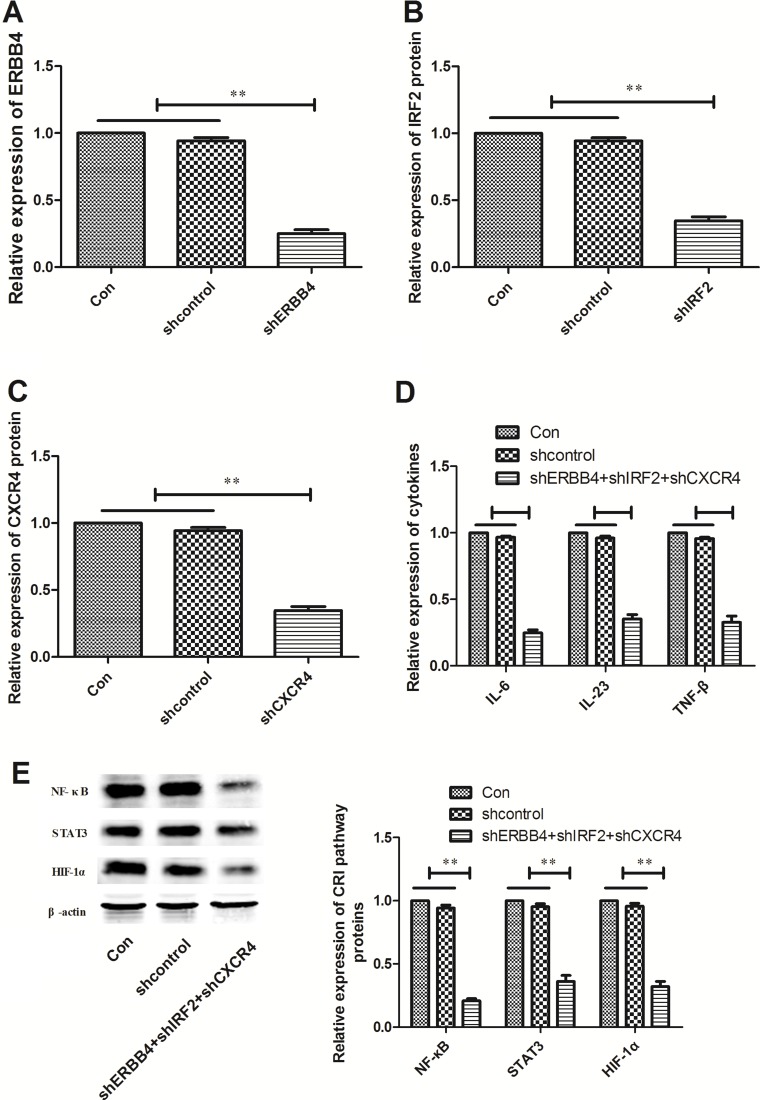
Effects of ERBB4, IRF2 and CXCR4 knockdown CRI critical pathway and cytokines (**A**) Western blot of ERBB4 protein expression in TE11 cells transfected with pGLV3/shXIAP or pGLV3/shcontrol, respectively. (**B**) Western blot of IRF2 protein expression in TE11 cells stably transfected with pGLV3/shXIAP or pGLV3/shcontrol, respectively. (**C**) Western blot of CXCR4 protein expression in TE11 cells stably transfected with pGLV3/shXIAP or pGLV3/shcontrol, respectively. (**D**) Effect of down-regulation of ERBB4, IRF2 and CXCR4 together on cytokines (IL-6, IL-23, and TNF-β) expression. (**E**) Effect of down-regulation of ERBB4, IRF2 and CXCR4 together on protein expression of NF-κB, STAT3, and HIF-1α. U6 or β-actin was used as an internal control. Each experiment was performed at least in triplicate. *P < 0.05, **P < 0.01 vs control.

ERBB4, IRF2 and CXCR4 were directly targeted by miR-302b and related to NF-κB, STAT3, and HIF-1α respectively, we hypothesized that miR-302b might regulate CRI of EC via down-regulation of these three proteins. In order to validate this hypothesis, pGLV3/shERBB4, pGLV3/shIRF2, pGLV3/shCXCR4 or pGLV3/ shcontrol was constructed and stably transfected into TE11 cells. Western blot confirmed the decreased expression of ERBB4, IRF2 and CXCR4 protein in TE11 cells compared with mock and TE11/shcontrols (Figure 5A–5C). Further experiments showed that inhibition of target genes together down-regulation also decreased the expression of IL-6, IL-23, and TNF-β (Figure 5D). Moreover, all of target genes inhibitation decreased NF-κB, STAT3, and HIF-1α expression in TE11 cells as the effect of up-regulation of miR-302b (Figure 5E).

### miR-302b inhibits tumor growth and CRI *in vivo*

**Figure 6:**
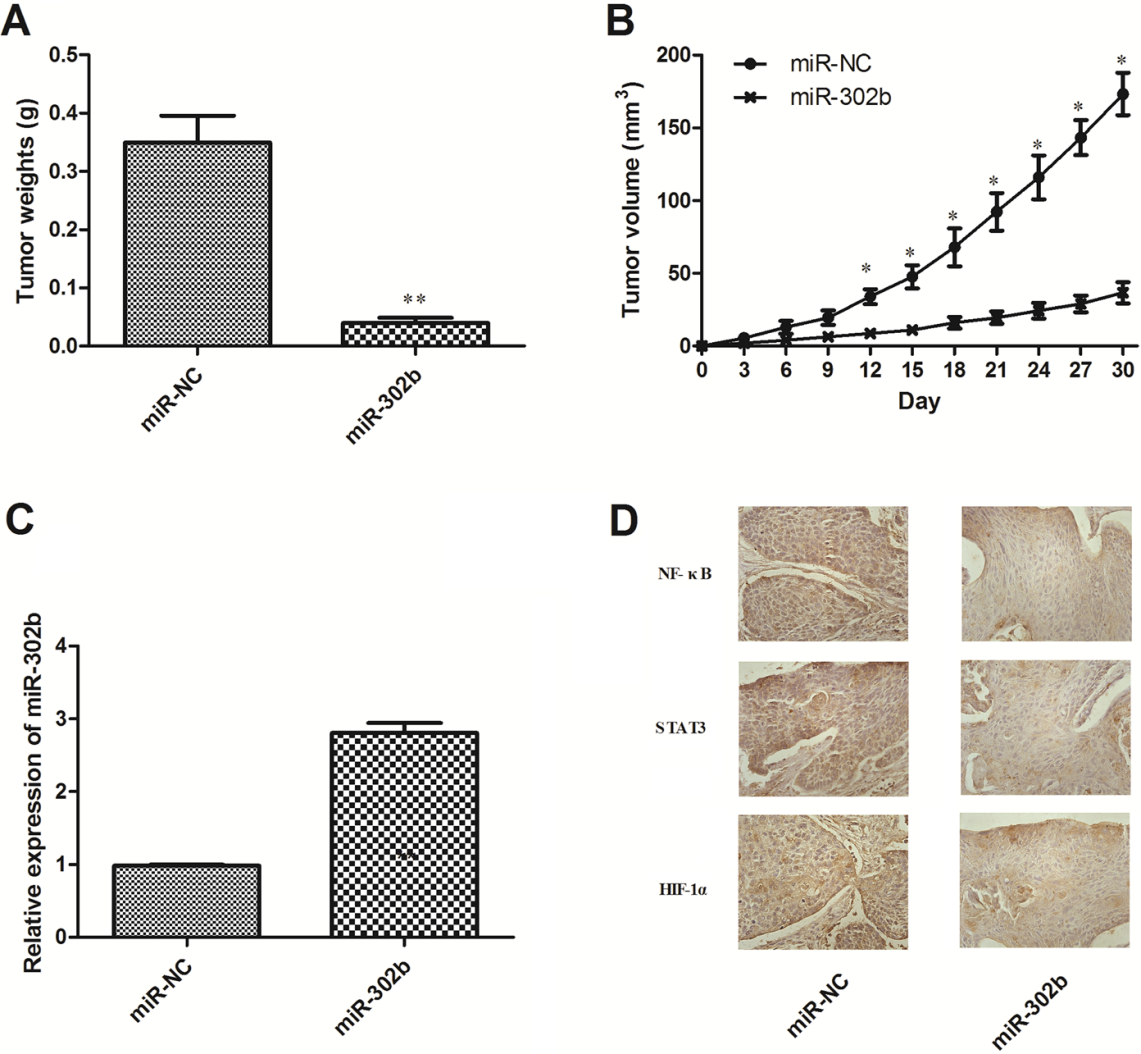
Effect of miR-302b on EC growth and CRI critical pathway *in vivo* (**A**) Tumor weight. (**B**) Tumor growth curves. (**C**) The expression levels of miR-302b were detected by qRT-PCR analysis in the tumor tissues from the animal. (**D**) IHC staining of NF-Κb/STAT3/HIF-1α in the tumor tissue from miR-NC and miR-302b injected mice. Each experiment was performed at least in triplicate. *P < 0.05, **P < 0.01 vs control.

miR-302b inhibited ESCC cells growth *in vitro* in our previous studies, so we then tested its effect *in vivo* xenograft model. miR-302b and miR-NC–transfected TE11 cells were injected subcutaneously into the posterior flank of nude mice. The average tumor weights for the miR-NC and the miR-302b groups on day 30 were 0.35 and 0.04 g, respectively (Figure 6A). And the average volume of miR-302b–treated tumors was smaller than that in miR-NC (Figure 6B). Furthermore, the expression levels of miR-302b in the tumor tissues were examined by qRT-PCR. Consistent with the *in vitro* data, the *in vivo* data showed that the expression of miR-302b was increased (Figure 6C). Immunohistochemical analysis also demonstrated decreased NF-κB, STAT3, and HIF-1α expression levels in the tumor tissue treated with miR-302b (Figure 6D). Moreover, the location of these proteins in miR-302b treated tissues was mainly in cytoplasm, while in control they were mainly in nucleus, suggesting CRI critical transcription factors inactivated under miR-302b treatment.

## DISCUSSION

Epidemiological studies has estimated that underlying infections and inflammatory responses are linked to 15–20% of all deaths from cancer worldwide [14]. On the other hand, an inflammatory component, which is not epidemiologically related to cancer, is also present in the microenvironment of tumors. With the progress on the relationship between inflammation and cancer, cancer-related inflammation (CRI) has been proposed as a major physiological hallmark of malignancy [15, 16].

The available experimental data have thrown new light on molecular and cellular mechanisms about inflammation and cancer: inflammation promoting cancer through common inflammatory mediators at the intersection of the intrinsic and extrinsic pathway including transcription factors, cytokines, and chemokines. Through different signaling pathways, CRI can affect many aspects of malignancy, including the proliferation and survival of malignant cells, angiogenesis, tumor metastasis, and tumor response to chemotherapeutic drugs and hormones in malignant cells including EC cells [17–20]. There ought to be a relationship between CRI and EC, especially for EAC. As one of the major risk factors for EAC, Barrett's esophagus was always caused by chronic inflammation associated with chronic reflux esophagitis [21]. For ESCC, there are also relationships with chronic inflammation-associated genomic instability [22]. As in our previous reviews, key mediators of CRI play an important role in the carcinogenesis and progression of EC, suggesting that reciprocal interaction between CRI and EC, but more experiments are needed to confirm the exact regulating mechanisms [5].

miRNAs can post-transcriptionally regulate gene expression and play important role in physiology and pathology progression. Moreover, one single miRNA effect lots of genes together, suggesting that they have the ability to control complex signal pathways including CRI. Lots of reports investigated the role of miRNAs in CRI, but studies of miRNAs in CRI in EC are quite limited [23, 24]. Our previous study found that miR-302b was a potential molecular marker and inhibited proliferation by inducing apoptosis and repressed invasion potentially by targeting ErbB4 in ESCC [7].

Thus, in this study we have evaluated a potential alteration in the expression of miR-302b in EC tissues, cell lines, and cell lines under inflammation stimuli. We found that miR-302b was significantly down-regulated in EC cell lines and tissues and miR-302b decreased under different inflammation stimuli. Then, over-expression of miR-302b decreased the expression of CRI critical pathway and cytokines, while down-expression of miR-302b got the opposite result. These data suggestted that miR-302b involved in the inflammation regulation in EC cells and inhibited the CRI pathway. Considering our previous research about the tumor suppressor role of miR-302b in ESCC and role of CRI in tumor, our data suggesting that miR-302b inhibited proliferation by inducing apoptosis and repressed invasion potentially by decreasing CRI.

Using the bioinformatics database, ERBB4, IRF2 and CXCR4 are predicted to be targets of miR-302b. And ERBB4, IRF2 or CXCR4 had cross-talk with HIF-1α, STAT3, and NF-κB respectively [11–13]. Moreover, all of them function as oncogenes in EC in previous reports [25–30]. Basing on above background, we hypothesis that miR-302b may repress CRI in EC by targeting multiple genes closely related to CRI critical pathway. Here, we demonstrated that miR-302b suppressed ERBB4, IRF2 and CXCR4 expression by binding directly to the 3′-UTR of these target genes, and an inverse correlation was observed between miR-302b and target genes protein expression in EC tissues. We showed that silencing ERBB4, IRF2 and CXCR4 together by RNAi inhibited CRI, which was similar to that of miR-302b over-expression. Meanwhile, *in vivo* experiments also confirmed that miR-302b inhibited growth of EC cells. Last, immunohistochemical analysis demonstrated decreased NF-κB, STAT3, and HIF-1α expression levels in the tumor tissue treated with miR-302b. These results may at least partially, explain the antitumor effects of miR-302b involved in EC.

## MATERIALS AND METHODS

### Patient samples

Between January 2015 and February 2015, 15 patients received resection for EC at Tangdu Hospital, Fourth Military Medical University. As a result, 15 patients were retrospectively reviewed. None of these 15 patients received neoadjuvant therapy before operation. Fresh cancer tissues and paired normal adjacent tissues (NAT) were obtained from these patients. The Institutional Ethics Committee approved this project and written informed consents were obtained from the patients.

### Cell lines

The EC cell lines (TE1, OE33, TE11 and Ec9706) and esaphagel normal cell line (Het-1A) were obtained from the Cell Bank of Shanghai (China) and cultured in RPMI 1640 medium supplemented with 10% fetal bovine serum (FBS), 100 units/mL penicillin, and 100 g/mL streptomycin at 37°C in a 5% CO2 incubator. To mimic inflammation environment, cells were cultured with RPMI1640 medium containing LPS (500 ng/ml), IL-6 (10 ng/ml), IFN-γ (50 ng/ml), TGF-β (5 ng/ml) and normal RPMI1640 medium respectively.

### Quantitative reverse transcription-PCR (qRT-PCR)

qRT-PCR was carried out using the PrimeScript® RT reagent Kit (Perfect Real Time) and a BioRad iQ5 Real-Time PCR Detection System. The reverse transcription reaction was carried out in a 20 μL volume with 1 μg total RNA. The reaction was incubated at 37°C for 15 min, then 85°C for 5 sec; 1 μL of the RT product was used in each PCR. The PCR cycling began with template denaturation at 95°C for 5 min, followed by 40 cycles of 95°C for 10 sec, 60°C for 20 sec, and 72°C for 20 sec. U6 snRNA levels were used for normalization. A control reaction without reverse transcriptase was included, and the lack of signal from this reaction ensured that there was no genomic DNA contamination. In addition, the final PCR products were resolved using agarose gel electrophoresis, and a single band of the expected size indicated the specificity of the reaction. The expression levels relative to U6 were calculated using the formula 2^−ΔΔCT^.

### Immunoblot analysis

For immunoblot analyses, 20 μg total proteins were electrophoresed on a 10% SDS-PAGE gel, transferred to PVDF membrane, blocked, and then incubated with primary antibody. The blots were then incubated with the corresponding horseradish peroxidase (HRP)-conjugated secondary antibody at room temperature for 2 h. Then, the membranes were visualized by exposure to X-ray film in dark following a chemiluminescence reaction using the enhanced ECL detection reagents (Amersham, Little Chalfont, Buckinghamshire, England) according to the manufacturer's instructions. Densitometry analysis was performed using the Scion Image software.

### Plasmid construction and cell transfection

The sequence of the precursor miR-302b was synthesized and cloned into the pcDNA^™^6.2-GW/EmGFP-miR expression vector (Invitrogen, Carlsbad, CA, USA). The ERBB4, IRF2 and CXCR4 3′-UTR target site sequence and the sequence containing the mutation of three bases in the miR-302b target site were synthesized and cloned downstream of the luciferase gene in the pmirGLO luciferase vector (Promega, Madison, WI, USA). All procedures were performed as previously described [31]. These vectors were named miR-302b, ErbB4-W, ErbB4-M, IRF2-W, IRF2-M, CXCR4-W and CXCR4-M respectively. All constructs were sequenced. The anti-miR-302b inhibitor (2′-O-methyl antisense oligonucleotide, Anti-miR-302b) and the anti-miR-inhibitors-Negative control (2′-O-methyl scrambled miRNA, Anti-NC) were purchased from AngRang Inc. (Xi'an, China). pGLV3/shERBB4, pGLV3/shIRF2, pGLV3/shCXCR4, and pGLV3/shcontrol were purchased from GenePharma (Shanghai, China). Cell transfection was performed using Lipofectamine 2000 (Invitrogen, Carlsbad, CA, USA) according to the manufacturer's protocol. Cells were transfected and selected on Blasticdin (Invitrogen, USA) at 5 ug/ml for 4 weeks. Total RNA and protein were prepared 48 h after stable transfection and were used for qRT-PCR or immunoblot analysis, respectively. The primer sequences were listed in Table 1.

**Table 1:** The primer sequence used in the research

Name	Sequence
miR-302b-F	5′-GATAAGTGCTTCCATGT-3′
miR-302b-R	5′-CAGTGCGTGTCGTGGAGT-3′
U6-F	5′-CTCGCTTCGGCAGCACA-3′
U6-R	5′-AACGCTTCACGAATTTGCGT-3′
ErBb4-F	5′-AGGAGTGAAATTGGACACAGC-3′
ErBb4-R	5′-TCCATCTCGGTATACAAACTGGT-3′
IRF2-F	5′-TGAAGTGGATAGTACGGTGAACA-3′
IRF2-R	5′-CGGATTGGTGACAATCTCTTG-3′
CXCR4-F	5′-GGATATAATGAAGTCACTATGGGAAAA-3′
CXCR4-R	5′-GGGCACAAGAGAATTAATGTAGAAT-3′
GAPDH-F	5′-ACCACAGTCCATGCCATCAC-3′
GAPDH-R	5′-TCCACCACC CTGTTGCTGTA-3′

### Elisa analysis

The quantification of cytokine levels from the cell culture mediums was performed by duplication using ELISA kits for IL-6, IL-23, and TNF-β (Biosource International, Inc, CA, USA), according to the manufacturer's instructions. In each case, the optical density of known standards was used to construct a calibration curve and the mean cytokine values ± SD were then calculated for each sample.

### Luciferase assay

The cells were co-transfected with pmirGLO -WT or pmirGLO -MT and miR-302b or mock (pcDNA™6.2-GW/EmGFP-miR). Luciferase activity was measured 24 h after transfection using the Dual-Glo luciferase assay system (Promega, Madison, WI, USA). All experimental protocols were performed according to the manufacturer's instructions. The normalized firefly luciferase activity (firefly luciferase activity/Renilla luciferase activity) for each construct was compared to that of the pmirGLO Vector no-insert control.

### Analysis of tumorigenicity *in vivo*

All experimental procedures involving animals were in accordance with the Guide for the Care and Use of Laboratory Animals and were performed according to the institutional ethical guidelines for animal experiment. Viable miR-302b and negative control (NC)-transfected TE11 cells were suspended in 100 mL PBS and then injected subcutaneously into the posterior flank of nude mouse at 4 to 5weeks of age. Tumor growth was examined every 3 days for 4 weeks. Tumor volume (V) was monitored by measuring the length (L) and width (W) with calipers and calculated with the formula (L × W^2^) × 0.5.

### Immunohistochemical staining and analysis

The Streptavidin- Peroxidase technique (Golden Bridge International: SP-9000) was used according the manufacturer's instruction. A sheep polyclonal antibody against NF-κ B, STAT3, or HIF-1α (Santa Cruz Biotechnology, Santa Cruz, CA, USA) was used at different dilutions. An irrelevant sheep antiserum served as a negative control. Two observers who were blinded to clinical features evaluated staining results independently and co-observed for a consensus when they were divergent with the method as described [32].

### Statistical analysis

The data are expressed as the mean ± standard deviation. Differences between groups were assessed using an unpaired, two-tailed Student's *t* test; *P* < 0.05 was considered significant.

## CONCLUSIONS

In summary, we investigated the roles of miR-302b and its targeted genes, ERBB4, IRF2 and CXCR4, in their control of CRI critical pathway and cytokines in EC pathologic processes. Our findings suggest that miR-302b may be a novel CRI regulating miRNA that inhibits CRI critical pathway and downstream cytokines expression through targeting ERBB4, IRF2 and CXCR4, resulting in decrease of tumor growth. Our findings highlight the functional association of miR-302b and their host genes, providing new insight into the regulatory network of CRI in EC and opening the possibility for future therapeutic interventions.

## Abbreviations

EC: Esophageal cancer
EAC: esophageal adenocarcinoma
ESCC: esophageal squamous cell carcinoma
CRI: cancer-related inflammation
miRNA: microRNA.
